# The Effect of L-Glutamine on Basal Albumen and Yolk Indices, and Albumen Amino Acids Composition

**DOI:** 10.3390/ani11123556

**Published:** 2021-12-14

**Authors:** Ewa Tomaszewska, Anna Arczewska-Włosek, Artur Burmańczuk, Renata Pyz-Łukasik, Janine Donaldson, Siemowit Muszyński, Sylwester Świątkiewicz

**Affiliations:** 1 Department of Animal Physiology, Faculty of Veterinary Medicine, University of Life Sciences in Lublin, 20-950 Lublin, Poland; 2 Department of Animal Nutrition and Feed Science, National Research Institute of Animal Production, 32-083 Balice, Poland; anna.arczewska@iz.edu.pl (A.A.-W.); sylwester.swiatkiewicz@iz.edu.pl (S.Ś.); 3 Department of Pharmacology, Toxicology and Environmental Protection, University of Life Sciences, 20-950 Lublin, Poland; artur.burmanczuk@up.lublin.pl; 4 Department of Food Hygiene of Animal Origin, Faculty of Veterinary Medicine, University of Life Sciences in Lublin, 20-950 Lublin, Poland; renata.pyz@up.lublin.pl; 5 School of Physiology, Faculty of Health Sciences, University of the Witwatersrand, Johannesburg 2193, South Africa; janine.donaldson@wits.ac.za; 6 Department of Biophysics, Faculty of Environmental Biology, University of Life Sciences in Lublin, 20-950 Lublin, Poland

**Keywords:** L-glutamine, laying hen, egg quality, egg albumen, amino acids

## Abstract

**Simple Summary:**

At present, with increased consumers’ focus on eating healthy, it is expected that egg protein content and amino acids profile are among the components of eggs that play critical roles in egg selection. Thus, this research investigated the effect dietary L-glutamine supplementation has on basal albumen and yolk indices as well as albumen protein amino acid profile. The study shows a potential role of L-glutamine supplementation for enhancing nutritional values of eggs by the decrease of albumen lipid content and the change of amino acid profile.

**Abstract:**

The current study tested the hypothesis that 1.0% dietary inclusion of L-glutamine (Gln), an non-essential amino acid that influences protein synthesis, can improve internal egg quality, including amino acids profile. Thirty-week-old Bovans Brown laying hens in their middle laying period were assigned to one of the two experimental groups (12 replicate cages, 2 hens/cage) with Gln in the form of alpha-ketoglutarate (10 g/kg) or without Gln inclusion. The experimental period lasted for 30 wks, from the 31st to the 60th week of age of hens, when eggs were collected and selected egg quality indices were determined. Gln supplementation had no effect on albumen and egg yolk share, albumen and yolk basal indices and composition, including yolk cholesterol content. However, Gln decreased the lipid content of the egg albumen (*p* < 0.001), and influenced albumen amino acid profile, increasing content of asparagine (*p* < 0.05), phenylalanine (*p* < 0.05), proline (*p* < 0.001), tryptophan (*p* < 0.01), and tyrosine (*p* < 0.05). In conclusion, the study shows a potential role of Gln supplementation for enhancing nutritional values of eggs by lower lipid content and higher amino acid profile.

## 1. Introduction

In the early 1950s, Kramer [1] defined the quality of any food as the properties influencing the acceptance or rejection of this food by the user, and despite this definition is over 60 years old, it still applies to majority of food products, including eggs. Egg quality as a general term refers to several features defining both its internal and external quality. In contrast to external egg quality, internal egg quality refers not only to the physical properties of the albumen (e.g., viscosity) and the egg yolk (e.g., color, strength of vitelline membrane), but primary to their functional properties [2,3]. Protein and amino acids are considered key components of each living organism [4]. Egg albumen consists of about 10% of protein, with many functionally important proteins, like ovalbumin (54%), ovotransferrin (12%), ovomucoid (11%), lysozyme (3.5%), and ovomucin (3.5%) [4]. Eggs have been identified as the lowest-cost source for proteins among all protein sources in the USA [5], and very useful sources of high-quality protein in developed and developing countries alike, with high biological value and digestibility [6]. Taking into account the increasing demand for proteins in the food industry related to both increasing population growth as well as increasing interest in egg components acting as nutraceuticals such antioxidant properties, antimicrobial activities, immunomodulatory, and anticancer important for a healthy lifestyle, it is expected that protein content and amino acids profile will become the components of eggs that could play critical roles in egg selection, especially that egg taste can be changed by altered contents of free amino acids [7,8,9].

L-glutamine (Gln) is considered a non-essential amino acid from the point of view of physiological role in general metabolism (including protein and lipid synthesis), however it is suggested that in some critical conditions like stress, malnutrition, rapid growth or egg production, it can act as conditionally essential amino acid [10,11,12,13,14,15,16,17]. Previous in vitro and animal models experiments, including those with both birds and mammals, have shown Gln to have a protective nutritive role, serving both as the main source of energy for enterocytes division and playing key role in the maintenance of the intestinal barrier [16,18,19,20,21,22]. Gln is widely used as a dietary additive in livestock animals, but as it is unstable in water solution and produces toxic byproducts (such as ammonia) on decomposition, to avoid these problems, its precursor alpha-ketoglutarate (AKG) is considered as a favorable way for providing Gln [16,18,19,20,22,23,24,25,26]. It is known that Gln is an energy donor and stimulator of protein synthesis, and it also modulate general metabolism of living organism, improve nitrogen balance, thus the growth and body weight gain. Another mechanism of Gln includes impact on the endocrine system, because Gln is transformed into ornithine and arginine, which stimulate the secretion of growth hormone and insulin-like growth hormone. Moreover, Gln given via the diet can influence microbial flora present in the gut and intestinal barrier [14,16,17,18,19,21].

In previous poultry studies Gln doses of 0.5, 1.0, 1.5, 2.0, and 4.0% were tested in relation to performance parameters. The most optimized effects in all poultry species were obtained for a concentration of 1.0% (10 g/kg) Gln or AKG supplementation. In broilers the dose of 1.0% improves growth performance, such as daily body weight gain, feed intake, feed conversion ratio, and slaughter yield; intestinal morphology, the rate of development and recovery after harmful factors; humoral immune response, or blood morphology [10,11,12,14,15,17]. Gholipour et al. [27,28] have reported that 1.0% Gln has the most positive effects on performance, egg quality traits, and fertility parameters in Guinea fowls; and Zavarize et al. [13] show that the dose of 1.0% improved feed intake, feed conversion per kilogram of eggs, and egg quality in laying hens. Our recent study shows positive effects of long-term 1.0% AKG supplementation in laying hens on meat quality, structure of articular cartilage as well as strong anti-osteoporotic action in cancellous and medullary bone [29]. AKG administered to laying hens has decreased cholesterol content in blood and breast muscles of laying hens, and serving as a substrate in fatty acid synthesis affected the fatty acid profile and cholesterol content of the egg yolk, thus influencing the nutritional value of the egg yolk [29,30].

While all these mentioned above experiments primarily focused on the physiological and nutritional consequences of dietary Gln as functional additive promoting growth and a few relate to performance or egg production, there is still a lack of information relating Glu supplementation to internal egg quality including amino acids profile.

Therefore, in this study, we hypothesized that Gln, showing so pleiotropic action, supplemented to laying layers at the dose selected on the welfare of poultry could improve basal albumen and yolk indices. Except for the verification of the above presented hypothesis, the study focused mainly whether Gln given to hens at a dose of 1.0%, which was selected from the point of view of hens welfare, for 30 weeks in middle birds’ peak lay period would affect the amino acid profile of the egg albumen.

## 2. Materials and Methods

### 2.1. Animals and Experimental Design

The experiment was carried out using 48, 17-wk-old, Bovans Brown hens. The birds were obtained from a local commercial source and housed in cages 30 × 120 × 50 cm (two birds per cage), on a wire-mesh floor, under controlled climate conditions (19 ± 2 °C; 50–65% rh; 14 L:10 D lighting schedule with light intensity of 10 lux), with free access to feed and water. During the pre-experimental period (from the 18th to 30th week of age), all hens were fed the same commercial pre-laying (18th–20th wk) and laying (21st–30th wk) diets. The experimental period lasted for 30 weeks (from the 31st to the 60th wk of age of the hens), and included the middle period of laying lasting from 18th to 100th wk of age. Selected time-span for supplementation stars after the peak of laying and finishes before time when the egg production does not decrease so much when economic demands indicate the necessity of reconstitution of the flock. Hens were randomly assigned to one of the two experimental groups, each compromising 12 replicate cages, with two birds per cage. Using our previously obtained results and data from the literature, as well as taking into account the 3R’s principle, we performed this study with one dose of Gln. The control group was fed the basal diet in the form of mash complete feed, and the experimental group (the Gln group) was fed the same basal diet with 1.0% (10 g/kg) of Gln. in the form of AKG (powder, Protista Biotechnology AB, Lund, Sweden) mixed with the basal diet intended for the Gln group. The composition and nutritive value of the basal diet are given in Table 1. Gln (10 g/kg feed) was administered in the complete diet and was not taken into account when balancing the compound feed. All the diets (pre-experimental and experimental) met the nutrient specifications according to the Polish-recommended requirements [31]. The chemical composition of the basal feed was determined for dry matter, crude protein, crude fat, crude fiber, and ash according to Commission regulation (EC) 152/2009 (Annex III point A, C, H, I, M, respectively) [32]. The Gln concentration in the basal diet was not determined. During the entire experiment period, feed intake and laying rate were recorded. From the eggs collected at the last two days of the study (at the end of 30th wk of the experiment), two eggs from each cage were randomly selected (*n* = 24 in each experimental group). All eggs were stored in a cold room (4 °C) after collection and analyzed within 3 days after collection.

### 2.2. Samples Preparation and Analysis

Eggs were cracked in order to transfer the internal contents to a glass plate, and the albumen height was measured using a tripod electronic height gauge that is part of the Technical Services and Supplies QDC Egg Quality System (TSS, York, UK), followed by the calculation of the Haugh unit (HU) [33]. Yolk color was determined with a 16-point Roche color fan (DSM, Heerlen, Netherlands). After separation of the components from one another, the weight of albumen and yolk were recorded and the proportions of yolk and albumen fraction were evaluated.

The composition of the egg yolk and albumen was determined according to the methods of AOAC International [34]. The moisture content was measured by drying the samples in an oven at 103 ± 2 °C for 24 h (method 934.01). Crude fat (ether extract) was determined using a Soxtec System HT2 1045 extraction unit (Tecator, Hoganas, Sweden), by following the procedure described in method 920.39. The protein content was estimated by the Kjeldahl method, with a Tecator Kjeltec System 1026 distilling unit (Tecator, Hoganas, Sweden), after acid digestion (method 954.01). The protein content was estimated using 6.25 as a conversion factor of nitrogen to crude protein. Ash was determined using a gravimetric method (method 942.05). The amino acid profile of the egg albumen was determined using an INGOS AAA400 apparatus (Ingos Corp., Prague, Czech Republic), for the automatic analysis of amino acids, using the AOAC method 994.12. The amino acid content in the samples (except for cystine, methionine, and tryptophan) was determined after acid hydrolysis with 6 N·HCl at 110 °C for 20 h [35]. Cystine and methionine were measured as cysteic acid and methionine sulphone, respectively, by performing acid oxidation before hydrolysis with 6 N·HCl [36]. Tryptophan content of the egg albumen was determined using ion exchange chromatography (method 988.15) after alkaline hydrolysis with Ba(OH)_2_ at 110 °C for 20 h [37]. Egg yolk cholesterol content was determined using gas chromatography (Model 3400, Varian Inc., Walnut Creek, CA, USA; temperature of FID detector: 300 °C, carrier gas: helium), according to the method described by Hwang et al. [38], using an internal standard (5-alpha-cholestan, Sigma-Aldrich, St. Louis, MO, USA).

### 2.3. Statistical Analysis

As replicate cage served as the experimental unit for all analyses, the values of each measured trait of individual egg in a cage were averaged per cage prior to statistical analysis (*n* = 12 per group). Sample size was calculated for a two-tailed Student’s *t*-test with an α of 0.05 and power at 0.8 [39]. Available literature data show that for these assumptions sample size of *n* = 12 has a power of 80% to detect a change of 16% in Haugh unit and a change of 10%, 16%, 17%, and 19% change in egg albumen glutamic acid, leucine, tyrosine, and arginine content, respectively, assuming a 5% significance level [40]. A Shapiro-Wilk normality test was applied to test normality of the data. The homogeneity of variances was assessed using the Levene’s test. Normally distributed variables were analyzed using a two-tailed Student’s *t*-test or *t*-test with Welch’s correction when data lacked equal variances; non-parametric data were analyzed using a Mann–Whitney U test. For all tests, a *p*-value < 0.05 was established as statistically significant. Effect size (ES) for all significant comparisons was estimated with Cohen’s d for parametric comparisons with pooled standard deviation, and Cohen’s r for non-parametric comparisons, by dividing the Z value by the square root of the sample size [41]. All statistical procedures were conducted using Statistica software (v. 13.3, TIBCO Software Inc., Palo Alto, CA, USA).

## 3. Results

Daily feed intake recorded during the entire experiment period was 118.8 g ± 2.5 and 117 g ± 2.1 feed per hen in the control and Gln group, respectively; while laying rate amounted 95.8% ± 0.9 and 96.6 ± 0.8 in the control and Gln group, and there was no difference between both groups.

All parameters related to various albumen and yolk indices were unchanged following Gln supplementation to laying hens for 30 weeks (starting at the age of 31 weeks) (Table 2; for all *p* > 0.05). Gln supplementation decreased the lipid content of the albumen (by 73%, ES: 3.61; *p* < 0.05), whereas no other albumen or yolk components were affected (*p* > 0.05; Table 3).

Dietary supplementation of Gln to laying hens resulted in an increase in the asparagine (5.0%, ES: 1.06; *p* < 0.05), phenylalanine (3.7%, ES: 0.89; *p* < 0.05), proline (8.5%, ES: 1.71; *p* < 0.001), tyrosine (2.89%, ES: 0.85; *p* < 0.05), and tryptophan (6.9%, ES: 1.961; *p* < 0.01) content of the albumen (Table 4). No other changes in amino acid profiles were observed.

## 4. Discussion

The egg serves as a source of nutrients for humans. More frequently the consumers’ preference relating pro-health properties, e.g., antioxidants, lipid content, have been taken into consideration [42]. However, in the past few years the relationship between eggs amino acids and taste (sweetness, sourness, saltiness, bitterness, and umami) has been studied [6]. Among these amino acids are essential and non-essential. Essential amino acids for humans are arginine, leucine, isoleucine, lysine, methionine, phenylalanine, threonine, tryptophan, and valine [43,44]. Our study showed that the most abundant amino acids in the albumen of the eggs were aspartic acid, serine, glutamic acid, valine, leucine, and lysine, while histidine was the most limiting amino acid. These results are in agreement with other reports, where the same amino acids were in the abundance, irrespective of the source of eggs [6]. The Gln supplementation to laying hens in the present study resulted in an increase in asparagine, proline, phenylalanine, tyrosine, and tryptophan content. Asparagine is a non-essential amino acid. AKG is a precursor not only of Gln, but also of proline and asparagine [45,46]. Asparagine is required for the development and function of the brain and it participates in protein synthesis [46]. Besides glycine and hydroxyproline, proline is the most abundant amino acid in collagen, which is essential in the maintenance of the normal structure and strength of bones, skin, cartilage, and blood vessels [44]. Proline is required for the development of elastin, another primary structural protein in connective tissue [47]. Phenylalanine is an essential amino acids and a precursor of tyrosine (amino acid also increased after Gln supplementation), neurotransmitters important for proper function of each living organism (dopamine, noradrenaline, and adrenaline) as well as melanin. Phenylalanine possesses anti-depressant potential. The milk of mammals and eggs are sources most abundant of phenylalanine. Tyrosine is a non-essential amino acid formed from phenylalanine. The thyroid hormones triiodothyronine (T3) and thyroxine (T4) in the thyroid are derived from tyrosine, which also serves as a precursor for neurotransmitters. Tryptophan is a precursor for serotonin and melatonin and participates in the modulation of the endocrine system and the regulation of appetite [48]. The increase in aspartic acid, proline, and tryptophan in the egg albumen, following Gln supplementation in the current study seems to be beneficial for the consumers of the eggs especially that free amino acids relate to food taste. Among taste-active amino acids are umami taste (glutamic acid, aspartic acid, alanine, and tyrosine), sweet taste (methionine, alanine, glycine, proline, serine, valine, and lysine), sour taste (aspartic acid, glutamic acid, and lysine), bitter taste (proline, glycine, valine, leucine, tyrosine, phenylalanine, histidine, lysine, isoleucine, arginine, and tryptophan), salty taste (asparagine), and astringent taste (lysine) [9]. However, one of the limitations of our study was the fact that the amino acid profile of the egg yolk was not determined.

Our study also showed that Gln given to laying hens decreased the lipid albumen. Fresh egg white contained lipids, and phospholipids for the lipids. They are made up of triglyceride, diglyceride, free fatty acid, cholesterol ester, and cholesterol in acetone-soluble materials, and phosphatidylcholine, lysophosphatidylcholine, phosphatidylethanolamine, sphingomyelin [49]. The lipid profile of the egg can be affected by the genetics and age of the hens, as well as through modification of the composition of the feed provided to the hens [3,50], however it relates to the lipid composition of the egg yolk. It is proven also that Gln supplementation affected the FA composition of the egg yolk [30], however the mechanism of the Gln effect on lipid albumen is not known and should be studied.

Although the present study has some limitations—such as being a part earlier study, using Gln, which was selected from the point of view of hens welfare, and therefore cannot be categorically considered as the most favorable in terms of its effect of egg quality in general, as well as using a one-time point in laying cycle in which eggs were analyzed —but in our opinion, the obtained results clearly showed a novel aspects of Gln supplementation to laying hens, i.e., changes amino acids profile in albumen in post-peak lying period. Nevertheless, despite the undoubtedly positive results, the presented study cannot be considered as final, as the dose selection was based on performance and the physiological indicators of poultry welfare, and it cannot be categorically stated that this dose can be considered as the most optimal dose in the context of egg production and quality. Although, it is known that the egg is plastic enough to be modified by the diet of laying hens, but further research are needed, in which hens welfare (including behavioral, physical, physiological and production oriented welfare indicators), performance, and eggs quality should be taken into consideration.

## 5. Conclusions

In conclusion, as shown in the current study, Gln supplementation had no effect on albumen and egg yolk share, albumen and yolk basal indices and composition, including yolk cholesterol content. However, Gln decreased the lipid content of the egg albumen and influenced the albumen amino acid profile, increasing content of asparagine, phenylalanine, tryptophan, and tyrosine.

## Figures and Tables

**Table 1:** Composition (g/kg) and nutritive value (g/kg) of the experimental diets (air-dry basis).

Ingredients	Content
Ingredient (g/kg)	
Corn	422.10
Wheat	210.00
Soybean meal	236.00
Rapeseed oil	20.00
Limestone	90.00
Monocalcium phosphate	12.50
NaCl	3.00
DL-Methionine	1.40
Vitamin-mineral premix ^1^	5.00
Chemical composition	
Metabolizable energy, MJ/kg ^2^	11.60
Dry matter ^3^	901.8
Crude protein ^3^	163.5
Crude fat ^3^	29.2
Crude fiber ^3^	22.2
Crude ash ^3^	116.8
Lysine ^4^	8.35
Methionine ^4^	4.10
Methionine + Cysteine ^4^	7.2
Tryptophan ^4^	2.0
Threonine ^4^	6.3
Isoleucine ^4^	4.5
Leucine ^4^	7.1
Valine ^4^	14.5
Phenylalanine + Tyrosine ^4^	8.0
Total calcium ^4^	37.00
Total phosphorus ^4^	6.15
Available phosphorus ^4^	3.90

^1^ The premix provided per 1 kg of diet: vitamin A, 10,000 IU (retinol); vitamin D3, 3000 IU (cholecalciferol); vitamin E, 50 IU (dl-alpha-tocopherol); vitamin K3, 2 mg (menadione); vitamin B1, 1 mg (thiamine); vitamin B2, 4 mg (riboflavin); vitamin B6, 1.5 mg (pyridoxine); vitamin B12, 0.01 mg (cyanocobalamin); Ca-pantotenate, 8 mg; niacin, 25 mg; folic acid, 0.5 mg; choline chloride, 250 mg; manganese, 100 mg; zinc, 50 mg; iron, 50 mg; copper, 8 mg; iodine, 0.8 mg; selenium, 0.2 mg, cobalt, 0.2 mg. ^2^ Calculated as a sum of the ME content of components. ^3^ Analyzed value according to methods described in Commission regulation (EC) [32]. ^4^ Calculated value from the chemical composition of raw feedstuffs.

**Table 2:** The effect of dietary L-glutamine (Gln) supplementation (1.0%) on albumen and yolk indices of eggs from laying Bovans Brown hens at the end of a 30-week-long supplementation period (31st–60th wks of age).

Item	Control	Gln	*p*-Value
Egg weight, g	60.8 ± 0.9	62.6 ± 0.5	0.104
Yolk fraction, %	27.0 ± 0.6	27.4 ± 0.6	0.646
Albumen fraction, %	60.3 ± 0.6	59.7 ± 0.7	0.519
Albumen height, mm	6.20 ± 0.22	5.66 ± 0.28	0.147
Haugh units	76.7 ± 1.5	71.4 ± 2.4	0.073
Yolk color	3.79 ± 0.13	3.67 ± 0.14	0.559
Yolk weight, g	16.7 ± 0.3	17.4 ± 0.4	0.159

Data are expressed as the ls means ± SEM (*n* = 12). Significance was established using a two-tailed Student’s *t*-test (normally distributed data), *t*-test with Welch’s correction (normally distributed data with unequal variances), or the Mann–Whitney test (for pairwise comparisons with at least one non-normally distributed dataset).

**Table 3:** The effect of dietary L-glutamine (Gln) supplementation (1.0%) on the composition of the albumin and yolk of eggs from laying Bovans Brown hens at the end of a 30-week-long supplementation period (31st–60th wks of age).

Item	Control	Gln	*p*-Value
Albumen Components			
Water, %	87.55 ± 0.34	87.41 ± 0.21	0.612
Protein, %	11.38 ± 0.17	11.62 ± 0.34	0.527
Lipid, %	0.25 ± 0.01	0.16 ± 0.01 *	0.012
Ash, %	0.71 ± 0.01	0.72 ± 0.01	0.607
Yolk Components			
Water, %	52.77 ± 0.28	52.35 ± 0.34	0.350
Protein, %	16.84 ± 0.25	16.70 ± 0.07	0.857
Lipid, %	27.50 ± 0.46	27.32 ± 0.35	0.606
Ash, %	1.94 ± 0.05	1.82 ± 0.03	0.069
Cholesterol, mg/g	11.7 ± 0.17	11.6 ± 0.11	0.471

Data are expressed as the ls means ± SEM (*n* = 12). Significance was established using a two-tailed Student’s *t*-test (normally distributed data), *t*-test with Welch’s correction (normally distributed data with unequal variances) or the Mann–Whitney test (for pairwise comparisons with at least one non-normally distributed dataset); * *p* < 0.05.

**Table 4:** The effect of dietary L-glutamine (Gln) supplementation (1.0%) on the albumen amino acid content (mg/g of egg albumen) of eggs from laying Bovans Brown hens at the end of a 30-week-long supplementation period (31st–60th wks of age).

Item	Control	Gln	*p*-Value
Alanine	6.34 ± 0.05	6.47 ± 0.10	0.238
Arginine	5.83 ± 0.04	5.93 ± 0.10	0.417
Asparagine	11.62 ± 0.09	12.2 ± 0.21 *	0.016
Cysteine	4.09 ± 0.06	3.97 ± 0.08	0.271
Glutamic acid	14.52 ± 0.19	14.55 ± 0.22	0.921
Glycine	3.69 ± 0.04	3.77 ± 0.07	0.357
Histidine	2.49 ± 0.03	2.54 ± 0.04	0.346
Isoleucine	5.15 ± 0.04	5.18 ± 0.08	0.759
Leucine	9.27 ± 0.09	9.43 ± 0.15	0.359
Lysine	7.60 ± 0.06	7.64 ± 0.13	0.827
Methionine	4.85 ± 0.04	4.78 ± 0.08	0.368
Phenylalanine	6.18 ± 0.05	6.41 ± 0.10 *	0.032
Proline	3.55 ± 0.02	3.85 ± 0.07 *	<0.001
Serine	7.65 ± 0.10	7.83 ± 0.12	0.234
Threonine	4.91 ± 0.05	4.98 ± 0.08	0.367
Tryptophan	5.01 ± 0.07	5.54 ± 0.13 *	0.002
Tyrosine	3.80 ± 0.03	3.91 ± 0.04 *	0.040
Valine	7.08 ± 0.07	7.25 ± 0.14	0.301
*Sum of means*	113.80	116.23	

Data are expressed as the ls means ± SEM (*n* = 12). Significance was established using a two-tailed Student’s *t*-test (normally distributed data), *t*-test with Welch’s correction (normally distributed data with unequal variances) or the Mann–Whitney test (for pairwise comparisons with at least one non-normally distributed dataset; * *p* < 0.05.

## Data Availability

The data presented in this study are available on request from the corresponding author.
